# RAPD assisted selection of black gram (*Vigna mungo* L. Hepper) towards the development of multiple disease resistant germplasm

**DOI:** 10.1007/s13205-016-0582-8

**Published:** 2017-04-07

**Authors:** B. Vishalakshi, B. Umakanth, Anirudh P. Shanbhag, Arindam Ghatak, Nitish Sathyanarayanan, M. S. Madhav, G. Gopala Krishna, Hari Yadla

**Affiliations:** 1 Biotechnology Section, Crop Improvement Division, Indian Institute of Rice Research (ICAR-IIRR), Hyderabad, India; 2 Nuclear Agriculture and Biotechnology Division, Bhabha Atomic Research Centre (BARC), Mumbai, India; 3 Agri Biotech Foundation, Rajendra Nagar, Hyderabad, India; 4 Biomoneta Research Private Limited, Centre for Cellular and Molecular Platforms, NCBS, (TIFR), Bengaluru, India; 5 National Center for Biological Sciences (NCBS), TIFR, Bengaluru, India; 6 Regional Research Station (RARS-PJTSAU), Warangal, India

**Keywords:** Mung bean yellow mosaic virus (MYMV), Urdbean leaf crinkle virus (UCLV), Powdery mildew, Wilt, *Vigna mungo* L. Hepper, Rapid amplification of polymorphic DNA (RAPD)

## Abstract

Black gram (*Vigna mungo* L. Hepper), is an extensively studied food crop which is affected by many abiotic and biotic factors, especially diseases. The yield potential of Black gram is shallow due to lack of genetic variability and biotic stress susceptibility. Core biotic stress factors include mung bean yellow mosaic virus (MYMV), urdbean leaf crinkle virus (UCLV), wilt (*Fusarium oxysporum*) and powdery mildew (*Erysiphe polygoni DC*). Although many studies determine resistant varieties to a particular disease, however, it is often complimented by low yield and susceptibility to other diseases. Hence, this study focuses on investigating the genetic relationships among three varieties and nine accessions of black gram having disease resistance to previously described diseases and susceptibility using random amplified polymorphic deoxyribonucleic acid (RAPD) markers. A total of 33 RAPD primers were used for diversity analysis and yielded 206 fragments. Number of amplified fragments ranged from two (OPN-1) to 13 (OPF-1). The highest similarity coefficient was observed between IC-145202 and IC-164118 (0.921), while lowest similarity was between PU-31 and IC-145202 (0.572). The genetic diversity obtained in this study along with disease analysis suggests PU31as a useful variety for the development of markers linked to MYMV, UCLV, wilt and powdery mildew resistance by marker-assisted back cross breeding and facilitates the production of crosses with multiple disease resistance.

## Introduction

*Vigna mungo* L. Hepper (Black gram) is an important source of protein for both humans and cattle. It is grown in the Indian subcontinent as well as in Southeast Asia particularly in Thailand, Australia, and other Asian and South Pacific countries. There is a great need for cultivating richer protein sources for cattle feed and human consumption which has lead to great interest in studying the diversity and pathology in black gram (Wani et al. 2013). It is also one of the central crop resources in Indian cuisine and occupies about 3.15 mha. On an average, the crop yields up to 275 kg/ha, despite the estimated yield potential to be about 844 kg/ha (Amanullah and Hatam 2000).

Studies have been done to delineate the genetic diversity of various pulses including black gram which succeeded in producing high yielding varieties in various regions across Asia. However, high genetic consistency and the absence of suitable ideotypes reinforce with deficient harvest and disease susceptibility has caused major constraints in achieving a higher yield (Vyas et al. 2016). Among various biotic factors affecting black gram yield, the main diseases which devastate the crops are mung bean yellow mosaic virus (MYMV), urdbean leaf crinkle virus (UCLV), powdery mildew (PM) and wilt (*Fusarium oxysporum*).

MYMV, is a whitefly (*Bemisia tabaci*) transmitted Gemini virus, which hampers resistant black gram breeding due to lack of uniform screening procedures and in numerous cases resistance is governed by recessive genes (Haq et al. 2010). In such circumstances, indirect selection employing molecular markers linked to resistance genes should be an effective approach as they enable marker-assisted selection (MAS) to overcome the inaccuracies in the field evaluation (Xu et al. 2012). Few attempts were made to search molecular markers linked to MYMV resistance in black gram by utilizing resistant sources like TU 94-2 and PU 19 through RAPD and ISSR analysis (Souframanien and Gopalakrishna 2004). UCLV is another virus which is ubiquitous in all commercially viable *Vigna* genus including *V. mungo,* and affects the crop in all stages especially in early stage which devastates total seed yield. In time, leaves become leathery and crinkled and lose the ability to perform normal physiological functions (Gautam et al. 2016).

Powdery mildew (PM) caused by *Erysiphe polygoni DC* and wilt (*Fusarium oxysporum*) are two diseases caused by fungi which affect black gram in high humid conditions and are easily transmitted through sporulation. They affect all the parts of the plants, however, leaves are affected initially and the morphology of the plant begins to change as the diseases suggest (Jayasekhar and Ebenezar 2016). Diversity among powdery mildew resistance was estimated against eight black gram (*V. mungo*) varieties from various geographic locations of Uttar Pradesh and Andhra Pradesh (India) applying forty decamer primers and generated a total of 249 RAPD fragments, of which 224 were polymorphic (Datta et al. 2012).

Previously, genetic diversity analysis among 20 black gram varieties was done by employing RAPD and ISSR markers indicating homology between all the 20 black gram varieties. RAPD markers are very useful due to their simplicity, low cost and high throughput capabilities (Kumari and Thakur 2014). In fact, RAPD technique has been applied to assess molecular polymorphism in mung bean (Datta et al. 2012), chickpea (Singh et al. 2014), pea (Handerson et al. 2014) and pigeon pea (Yadav et al. 2014). Studies among the 20 black gram varieties showed that WBU-108 and RBU-38 were highly divergent, whereas LBG-648 and LBG-623 were genetically similar (Karuppanapandian et al. 2010). Genetic diversity analysis was performed on existing cultivars, to determine whether a lack of genetic variability might be a constraining factor to improvement (Sivaprakash et al. 2004). Host-plant resistance is considered as the most economical and eco-friendly strategy for achieving disease resistance and yield stability.

Current study focuses on predicting crosses between high yielding varieties and accessions which can incorporate resistance to the mentioned diseases. Not only have we chosen the varieties based on varying disease resistance but also taken into account the economic viability of these crops. We carried out genetic diversity analysis among different disease resistant and susceptible varieties utilizing 38 primers. Here is an emerging need to identify nucleic acid markers linked to disease resistance especially in staple crops such as black gram for preventing food deficit in a burgeoning population. The marker can be converted to sequence characterized amplified region (SCAR) to identify the locus of interest by amplification with a specific primer pair it could facilitate marker-assisted selection for rapid evaluation.

## Materials and methods

### Plant materials

Fifteen black gram varieties with varying disease resistance and susceptibility was obtained from Regional Agriculture Research Station, Guntur, India and National Bureau of Plant Genetic Resources, India (Table 1) which are distributed across northern (sub-tropical conditions) and southern parts of Indian subcontinent (tropical conditions).

**Table 1:** Black gram genotypes resistant and susceptible to various diseases (Srivastava et al. and Obiah et al.)

Disease	Resistant strains	Susceptible strains
Mung bean yellow mosaic virus (MYMV)	PU 1075, PU 31, PU 205	LBG 623, LBG 645, LBG 685, IC 110790, IC 145202, IC 1575, IC 164118, IC 20880, IC 59718, IC 61106, IC 61603, IC 73306
Powdery mildew (PM)	LBG 623, LBG 645, IC 145202, IC 1575, IC 59718, IC 61106, PU 31	LBG 685
Urdbean leaf crinkle virus (UCLV)	IC 73306	LBG 645, LBG 685
Wilt (*Fusarium oxysporum*)	LBG 623, LBG 645, LBG 685	IC 164118, IC 20880, IC 59718,

### Quantity and quality of genomic DNA

Genomic DNA from the obtained genotypes was isolated using a modified protocol prescribed by Saghai-Maroof et al. (1984). Final concentration of 30 ng/µl of genomic DNA (initial concentration ~100 ng/µl) was used for PCR (Thermo scientific). Initial denaturation conditions were 94 °C for 4 min, further continued for 1 min, annealing was at 33 °C and elongation was done at 72 °C for 1 min. Final elongation conditions were held for 10 min at 72 °C. PCR amplified products were subjected to electrophoresis on 1% agarose gel in 1XTBE buffer. Gel documentation was done using Alpha imager system.

Initially, 40 RAPD primers were tested on a subset of six black gram varieties and nine accessions. These primers were synthesized by Sigma-Aldrich and the sequences were used in previous studies on disease resistance in black gram (Basak et al. 2004; Vyas et al. 2016). Of the 40, only 33 RAPD primers produced clear and reproducible banding patterns. A total of 206 alleles were generated for the 33 selected RAPD primers and this allelic data was converted into binary data for identity matrix based on their presence or absence of alleles in terms of scores 1 and 0, respectively.

### Data analysis

Allele number was given and scored according to its presence or absence, based on difference in molecular weight. Only the clear and unambiguous bands were scored. Markers (33 RAPD primers) were scored for the presence (1) and absence (0) of the corresponding band among the genotypes. Consequently, a data matrix comprising ‘1’ and ‘0’ was formed and subjected to further analysis.

Further processing of data was done by carrying out sequential agglomerative hierarchial non-overlapping clustering (SAHN), on squared Euclidean distance matrix. Similarity matrix was done using Jaccard’s coefficient (Table 2) in which similarity matrices were utilized to construct the UPGMA (unweighted pair group method with arithmetic average) dendrogram. Data analysis was done using NTSYs PC (Numerical Taxonomy System, version 2.02).

**Table 2:** Primers used for the studying disease resistance in *Vigna mungo* genotypes

S. no.	Name	Primer sequence	Tm
1	OPN1	CTCACGTTGG	20.88
2	OPN2	ACCAGGGGCA	34.94
3	OPN3	GGTACTCCCC	22.41
4	OPN4	GACCGACCCA	29.8
5	OPN5	ACTGAACGCC	24.88
6	OPN6	GAGACGCACA	20.34
7	OPN7	CAGCCCAGAG	25.29
8	OPN8	ACCTCAGCTC	16.65
9	OPN9	TGCCGGCTTG	38.73
10	OPN10	ACAACTGGGG	24.06
11	OPN11	TCGCCGCAAA	40.01
12	OPN12	CACAGACACC	11.5
13	OPN13	AGCGTCACTC	18.1
14	OPN14	TCGTGCGGGT	36.84
15	OPN15	CAGCGACTGT	19.87
16	OPN17	CATTGGGGAG	25.36
17	OPQ1	GGGACGATGG	29.62
18	OPQ2	TCTGTCGGTC	18.18
19	OPQ3	GGTCACCTCA	17.53
20	OPQ4	AGTGCGCTGA	27.07
21	OPQ5	CCGCGTCTTG	33.18
22	OPQ6	GAGCGCCTTG	32.02
23	OPQ8	CTCCAGCGGA	31.46
24	OPQ9	GGCTAACCGA	26.67
25	OPQ16	AGTGCAGCCA	26.54
26	OPF1	ACGGATCCTG	23.93
27	OPF2	GAGGATCCCT	20.83
28	OPF3	CCTGATCACC	17.7
29	OPF4	GGTGATCAGG	17.7
30	OPH 3	AGACGTCCAC	16.83
31	OPH 6	ACGCATCGCA	33.99
32	OPH 7	CTGCATCGTG	22.22
33	OPH 8	GAAACACCCC	23.58
34	OPH 11	CTTCCGCAGT	25.64
35	OPH12	ACGCGCATGT	32.54
36	OPH15	AATGGCGCAG	32.75
37	OPH18	GAATCGGCCA	31.64
38	OPJ15	TGTAGCAGGG	21.52

## Results and discussion

Unraveling genetic diversity is quintessential to plant breeding, as the development of new varieties depends on the existing diversity of parent genotypes. This study focuses on understanding the crux of producing multiple disease resistant crop variety which can replace single disease resistant variety having respectable crop yield.

**Fig. 1:**
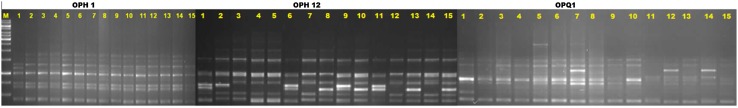
RAPD amplification pattern of black gram genotypes with primer OPH3, OPH12 and OPQ1. *Lane*-*M*–*1* *kb* marker, *lanes 1*–*15* indicate PU31, PU205, PU1075, LBG 623, LBG 645, LBG 685, IC-1575, IC-20880, IC-59718, IC-61603, IC-61106, IC-73306, IC-110790, IC-145202 and IC-164118

**Fig. 2:**
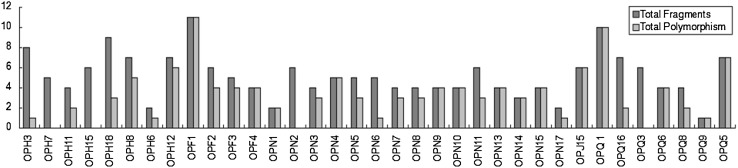
Bar graph showing various fragments produced by primers and total polymorphisms produced by them

DNA-based markers are effective and reliable tools for measuring genetic diversity in crop germplasm and studying evolutionary relationship. Molecular genetic techniques using DNA polymorphism is increasingly used to characterize and identify a novel germplasm for uses in the crop breeding process (Collard and Mackill 2008). Most markers produced polymorphisms, with the exception of OPH3, OPH12 and OPQ1 (Fig. 1) which showed monomorphic bands. Percentage polymorphism was calculated by considering the amount of polymorphism produced per fragment. OPF1, OPF4, OPN1, OPN4, OPN9, OPN10, OPN13, OPN14, OPN15,OPJ15, OPQ1, OPQ6, OPQ9, OPQ5 showed 100% polymorphism, where OPF1 generated maximum number of fragments (Fig. 2).

**Fig. 3:**
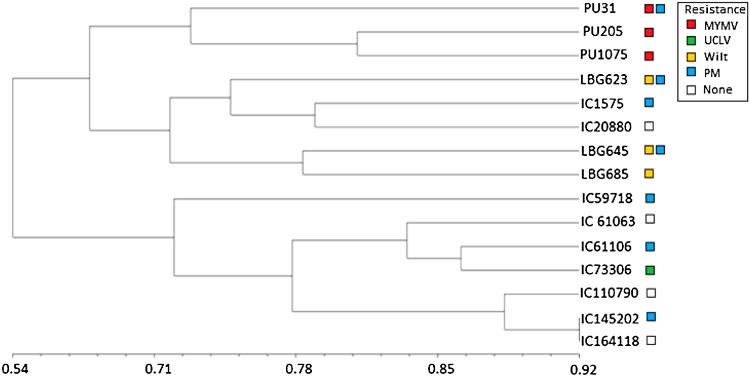
Dendogram of disease resistant and susceptible varieties, along with nine accessions using Jaccard’s similarity coefficient and UPGMA clustering. (*MYMV* mung bean yellow mosaic virus, *UCLV* urdbean leaf crinkle virus, *PM* powdery mildew)

After performing SAHN clustering on the binary matrix with subsequent calculation of Jaccard’s similarity dendrogram, the major cluster of 15 genotypes was divided into two sub-clusters, i.e., cluster I and cluster II at 64% of similarity. Cluster I accommodated PU-31, PU-205, PU-1075, LBG-623, IC-1575, IC-20880, LBG-645 and LBG-685, while cluster II comprised IC-59718, IC-61063, IC-61106, IC-73306, IC-110790, IC-145202 and IC-164118. Cluster I was further divided into two sub-clusters, i.e., Cluster Ia and Cluster Ib at 66% genetic similarity. Cluster Ia included three varieties, namely PU-31, PU-205 and PU-1075 displayed 74% similarity with remaining samples of cluster Ib. Cluster Ib was further divided into two clusters Ib_1_ and Ib_2_ at 73% of similarity. Cluster Ib_1_ had one MYMV susceptible variety (LBG-623) and two MYMV susceptible accessions, i.e., IC-1575 and IC-20880 and displayed 76% similarity with remaining samples of cluster Ib_2_. Ib_2_ accommodated two MYMV susceptible varieties, i.e., LBG-645 and LBG-685 which displayed 79% similarity with all the remaining samples in the cluster. Cluster II was further divided into two sub-clusters, i.e., IIa and IIb at 77% of similarity. The sub-cluster IIa contained one accession, i.e., IC-59718 which displayed 74% similarity with all remaining samples of cluster IIb and sub-cluster IIb was further divided into two clusters IIb_1_ and IIb_2_. IIb_1_ cluster displayed three accessions, i.e., IC-61063, IC-61106 and IC-73306 displayed 77% similarity with remaining samples of cluster IIb2 cluster. Cluster IIb_2_ showed three accessions IC-110790, IC-145202 and IC-164118 displayed 88% similarity with all the remaining samples in the cluster (Fig. 3).

Dendrogram analysis revealed that black gram accessions (IC-145202) and (IC-164118) had equal similarities, thus could not be used in hybridization process. Earlier, genetic diversity in black gram was studied using multivariate techniques on the basis of agronomic characters and identified the best genotypes for breeding (Ghafoor et al. 2001). Studies on genetic relationships between wild and cultivated *Vigna* species were done by cluster analysis and genetic distance determination using RAPD polymorphic markers (Sudha et al. 2014). Our studies clearly showed that the chosen varieties could be used for hybridization process.

This result indicates that there is a presence of great genetic variability among elite genotypes of black gram. The IC-164118 and IC-145202 showed very minimum differences and the maximum difference showed between PU-31 variety and IC-145202 accession at genotypic levels (Fig. 3).

Analysis of the relationship based on 33 RAPD markers revealed genetic diversity among the three resistant varieties and three susceptible varieties along with nine accessions. All the black gram samples were distinguished from one another based on these polymorphic bands. A dendrogram was prepared based on identity matrix taking into account the presence (1) or absence (0) of the bands. These coefficients have divided into two major clusters ranged from 0.64 to 0.92. The similarity coefficient was higher between IC-145202 (PM resistant) and IC-164118 (Disease susceptible) was 0.921 indicated less divergence between them. Lower similarity was observed between PU-31 (Resistant to MYMV and PM) and IC-145202 was 0.572 indicating high divergence.

Additionally, one RAPD marker, i.e., OPH-3 showed a resistance specific band in PU-31(MYMV resistant variety) and absent in remaining genotypes (Fig. 1. OPH-3 marker), this marker might be linked to MYMV resistance and can be useful for allele-specific sequence characterized amplicon region primer (SCAR). Although PU201 and PU1075 have phenotypically showed resistance to MYMV, but we did not get the expected band (1300 bp) in PU201 and PU1075, which indicates that there may be another new gene(s) showing resistance to MYMV in PU201 and PU1075. Hence, we suggest PU31 for producing disease resistant crosses in black gram genotypes.

This study could potentially identify diverse genotypes like PU 31(resistant to MYMV and PM) and IC-164118 (susceptible to MYMV and PM) for their use in the breeding program, further PU31 with LBG685 can yield resistance to MYMV, PM and wilt, while PU31 with IC73306 can yield resistance to MYMV, PM and UCLV. RAPD markers are useful in the assessment of black gram diversity, through detection of duplicate samples in germplasm collection, and the selection of a core collection to enhance the efficacy of germplasm management for use in black gram breeding and conservation programs. The genetic diversity obtained in this study might be useful in future strategies for the evolution of desired genotypes.

## Conclusion

Analysis of genetic diversity has aided in producing new varieties of a crop of interest, which are either high yielding or disease resistant. However, it has been traditionally confined to a particular disease and not a set of diseases which affect the crop. Since the advent of manufacture of disease resistant varieties in black gram research has focussed on making high yielding varieties which are also disease resistant. This has lead to multiple strains which have best of both worlds; however, producing a variety which is resistant to multiple diseases has always been a challenge. Our results show that significant divergence in black gram varieties has occured due to genesis of multiple breeding programs across agricultural institutes around the world. Our study has exploited this phenomenon and predicted which crosses are actually resistant to multiple diseases.

We have considered top disease factors which affect the yield of *V. mungo* to establish better crosses and improve crop production. Although we cannot predict a cross which can be resistant to all the four diseases at present, we are currently in the process of hybridizing and analyzing genetic diversity of the crosses and predicting a completely novel disease resistant black gram variety.
